# Adaptation and Coping Among Rural Older Adults Throughout the COVID-19 Pandemic

**DOI:** 10.1093/geroni/igab046.1618

**Published:** 2021-12-17

**Authors:** Andrea Huseth-Zosel, Heather Fuller

**Affiliations:** 1 North Dakota State University, Fargo, North Dakota, United States; 2 North Dakota State university, Fargo, North Dakota, United States

## Abstract

In the past year, older adults have faced challenges due to COVID-19, yet many have also shown great resilience. This qualitative study explores older adults’ experiences and perceptions of adaptation, social connection, and coping across the first six months of the COVID-19 pandemic, with a particular focus on unique resilience factors among rural older adults. A Midwestern sample (35% rural) of 70 older adults aged 70-97 completed three phone interviews (April, June, and October 2020) about their experiences with social distancing due to COVID-19. Thematic analysis of qualitative responses identified themes of resilience including: 1) purposeful and flexible social connections, 2) positive psychological mindset, and 3) hardiness and life experience. Strains related to the loss of community connections were evident, yet older adults demonstrated signs of adaptability and coping as compensation. Implications and future directions will be discussed in the context of change over time and geographic variation.

